# Supplementary material for: Single-step retrosynthesis prediction by leveraging commonly preserved substructures
Source: Nat Commun. 2023 Apr 28;14:2446. doi: 10.1038/s41467-023-37969-w (PMC10147675; doi:10.1038/s41467-023-37969-w)
Supplement: Supplementary file 1 — Supplementary Information [file 41467_2023_37969_MOESM1_ESM.pdf]

Supplementary Information for "Single-step Retrosynthesis Prediction by Leveraging Commonly Preserved Substructures"

Lei Fang[1*], Junren Li[2], Ming Zhao[3], Li Tan[4] and Jian-Guang Lou[1]

[1]Microsoft Research Asia, No.5 Dan Ling Street, Beijing, China
[2]College of Chemistry and Molecular Engineering, Peking University, No.5 Yiheyuan Road, Beijing, China
[3]IPS, Waseda University, 2-7 Hibikino, Wakamatsu-ku, Kitakyushu-shi, Fukuoka 808-0135, Japan
[4]Mincui Therapeutix, No.1 Yongtaizhuang North Road, Beijing, China
[*]Corresponding author, leifa@microsoft.com

Supplementary Methods

Pair-wise Ranker

SMILES strings of the predicted reactant fragments were decoded with the Transformer decoder using the beam search algorithm, a common search strategy for sequence-to-sequence learning models in natural language processing. The algorithm considers multiple options based on the beamwidth using conditional probabilities, and it usually performs better than the greedy search strategy. Similar to [1], we also discovered that some of the generated SMILES sequences were incorrect or could be different representations of the same SMILES.

We first explain how the final ranking is obtained in Transformer-based approaches [1], and then introduce the details of the pair-wise ranking model. Supplementary Figure 1 shows an illustrative example of the retrosynthesis prediction for a product with two substructures, each of which is augmented with 2 random SMILES. $\mathrm{f}(k)$ is a scoring function for entry at position k,

$$\mathrm{f}(k) = 1/(1 + \alpha * k) \tag{1}$$

where α is a hyperparameter and can be empirically set. For the example in Supplementary Figure 1, we obtained the ranking scores based on the predictions of sub_1 and sub_2 after incorrect and duplicated SMILES were removed (Phase 2 in Supplementary Figure 1), as shown in Supplementary Table 1. The final score for ranking was calculated by the sum of scores using sub_1 and sub_2, e.g., the score of p_3 was defined as $\mathrm{f}(1) + \mathrm{f}(3) + \mathrm{f}(1)$.

The value of α in the score function can affect the final ranking; it is tuned on the test set or empirically set [1], which motivates us to train the ranking model. We defined 4 types of features: the frequency, the percentage of rankings among top 1 and top 2, and the average ranking. These features were calculated among the predictions of unique substructures and all substructures. Supplementary

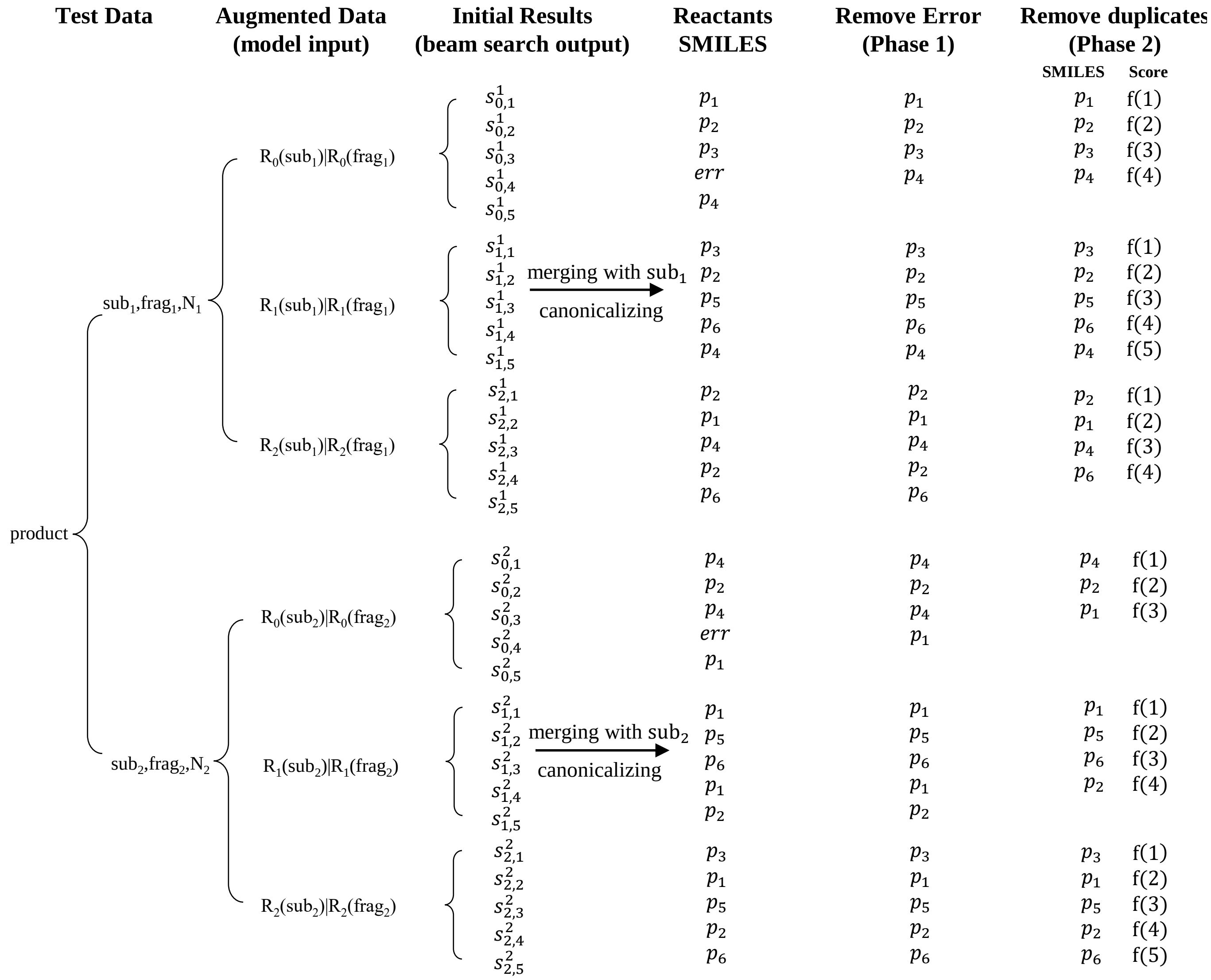

Supplementary Figure 1: Predictions for a product with two substructures. We show top 5 predictions for each beam. sub_i is the substructure extracted from N_i retrieved candidates. The product can be represented by the substructure sub_1 and fragment frag_1 or sub_2 and frag_2. R_0 is the function to obtain canonicalized SMILES, R_1 and R_2 are to obtain augmented random SMILES, $s^i_{j,k}$ is the k-th predicted fragments with beam search using $\mathrm{R}_j(\mathrm{sub}_i)|\mathrm{R}_j(\mathrm{frag}_i)$ as model input. We merge the predicted fragments $s^i_{j,k}$ with sub_i to obtain the molecules of reactants. After canonicalizing the SMILES and removing errors, we obtain the predictions in Phase 1 (denoted by p). We further filter out duplicated entries to obtain the predictions in Phase 2. $\mathrm{f}(k)$ is a scoring function for entry at position k.

SMILES	score using sub_1	score using sub_2
p_1	f(1) + f(2)	f(1) + f(2) + f(3)
p_2	f(1) + 2f(2)	f(2) + 2f(4)
p_3	f(1) + f(3)	f(1)
p_4	f(3) + f(4) + f(5)	f(1)
p_5	f(3)	f(2) + f(3)
p_6	2f(4)	f(3) + f(5)

Supplementary Table 1: Ranking scores using sub_1 and sub_2.

Table 2 shows the calculated features given the example in Supplementary Figure 1. As the generated SMILES sequences could be different representations of the same SMILES, we only used the highest ranking to calculate the average ranking. For example, among predictions of all substructures with incorrect SMILES removed (Phase 1), the ranking of p_1 on sub_1 and sub_2 is $\{1, 2\}$ and $\{4, (1, 4), 2\}$, respectively. $(1, 4)$ means that p_1 is predicted at position 1 and 4 in the same beam. The frequency of p_1 is $N_1 * 2_1 + N_2 * 4_2$, where N_1 and N_2 denote the number of candidates with sub_1 and sub_2 extracted as substructures, respectively. For all the feature values, the subscripts $_1$ and $_2$ correspond to sub_1 and sub_2, respectively. The average rankings for p_1 among sub_1 and sub_2 are $1.5(=\frac{1+2}{2})$ and $\frac{7}{3}(=\frac{4+1+2}{3})$, respectively. As p_1 was predicted by all substructures, its overall average ranking is $r_1 * (1.5)_1 + r_2 * (\frac{7}{3})_2$, where r is the percentage for each substructure, i.e., $r_1 = \frac{N_1}{N_1+N_2}$, $r_2 = \frac{N_2}{N_1+N_2}$ [1]. When duplicated entries are removed (Phase 2), the ranking of p_1 on sub_1 and sub_2 is $\{1, 2\}$ and $\{3, 1, 2\}$, respectively. The average ranking of p_1 on sub_1 is unchanged, and on sub_2 is changed to $2(=\frac{3+1+2}{3})$. Therefore, the overall average ranking of p_1 on Phase 2 is $r_1 * (1.5)_1 + r_2 * (2)_2$. Similarly, we calculated the features on predictions of unique substructures (Phase 2). For each predicted SMILES strings, we combined the features and obtained a 12-dimension vector as input to train the ranking model. The ranking model was a neural network with three linear layers, and the training objective was to ensure that golden reactants had a higher score than incorrect predictions.

Supplementary Results

Improvements by the pair-wise ranker

We introduce the pair-wise ranker to rerank the predictions. Here, we compare the results from using the score function in Augmented Transformer [1].The hyperparameter α is set to 3, which is carefully tuned on the test data. Supplementary Table 3 shows the comparison results on all test data and

[1]For predictions shown in Supplementary Figure 1, they occur in both substructures, thus the average ranking can be calculated by $r_1 * (\text{avg. rank})_1 + r_2 * (\text{avg. rank})_2$. If the SMILES is only predicted by sub_1, its average ranking will be $(\text{avg. rank})_1$.

SMILES	frequency	top 1 percentage	top 2 percentage	overall average ranking
Predictions of all substructures with incorrect SMILES removed (Phase 1)				
p_1	$N_1 * 2_1 + N_2 * 4_2$	$r_1 * (\frac{1}{3})_1 + r_2 * (\frac{1}{3})_2$	$r_1 * (\frac{2}{3})_1 + r_2 * (\frac{2}{3})_2$	$r_1 * (1.5)_1 + r_2 * (\frac{7}{3})_2$
p_2	$N_1 * 4_1 + N_2 * 3_2$	$r_1 * (\frac{1}{3})_1 + r_2 * 0_2$	$r_1 * 1_1 + r_2 * (\frac{1}{3})_2$	$r_1 * (\frac{5}{3})_1 + r_2 * (\frac{11}{3})_2$
p_3	$N_1 * 2_1 + N_2 * 1_2$	$r_1 * (\frac{1}{3})_1 + r_2 * (\frac{1}{3})_2$	$r_1 * (\frac{1}{3})_1 + r_2 * (\frac{1}{3})_2$	$r_1 * (2)_1 + r_2 * (1)_2$
p_4	$N_1 * 3_1 + N_2 * 2_2$	$r_1 * 0_1 + r_2 * (\frac{1}{3})_2$	$r_1 * 0_1 + r_2 * (\frac{1}{3})_2$	$r_1 * (4)_1 + r_2 * (1)_2$
p_5	$N_1 * 1_1 + N_2 * 2_2$	0	$r_1 * 0_1 + r_2 * (\frac{1}{3})_2$	$r_1 * (3)_1 + r_2 * (2.5)_2$
p_6	$N_1 * 2_1 + N_2 * 2_2$	0	0	$r_1 * (4.5)_1 + r_2 * (4)_2$
Predictions of all substructures with incorrect and duplicated SMILES removed (Phase 2)				
p_1	$N_1 * 2_1 + N_2 * 3_2$	$r_1 * (\frac{1}{3})_1 + r_2 * (\frac{1}{3})_2$	$r_1 * (\frac{2}{3})_1 + r_2 * (\frac{2}{3})_2$	$r_1 * (1.5)_1 + r_2 * (2)_2$
p_2	$N_1 * 3_1 + N_2 * 3_2$	$r_1 * (\frac{1}{3})_1 + r_2 * 0_2$	$r_1 * 1_1 + r_2 * (\frac{1}{3})_2$	$r_1 * (\frac{5}{3})_1 + r_2 * (\frac{10}{3})_2$
p_3	$N_1 * 2_1 + N_2 * 1_2$	$r_1 * (\frac{1}{3})_1 + r_2 * (\frac{1}{3})_2$	$r_1 * (\frac{1}{3})_1 + r_2 * (\frac{1}{3})_2$	$r_1 * (2)_1 + r_2 * (1)_2$
p_4	$N_1 * 3_1 + N_2 * 1_2$	$r_1 * 0_1 + r_2 * (\frac{1}{3})_2$	$r_1 * 0_1 + r_2 * (\frac{1}{3})_2$	$r_1 * (4)_1 + r_2 * (1)_2$
p_5	$N_1 * 1_1 + N_2 * 2_2$	0	$r_1 * 0_1 + r_2 * (\frac{1}{3})_2$	$r_1 * (3)_1 + r_2 * (2.5)_2$
p_6	$N_1 * 2_1 + N_2 * 2_2$	0	0	$r_2 * (4)_1 + r_2 * (4)_2$
Predictions of unique substructures with incorrect and duplicated SMILES removed (Phase 2)				
p_1	$2_1 + 3_2$	$0.5 * (\frac{1}{3})_1 + 0.5 * (\frac{1}{3})_2$	$0.5 * (\frac{2}{3})_1 + 0.5 * (\frac{2}{3})_2$	$0.5 * (1.5)_1 + 0.5 * (2)_2$
p_2	$3_1 + 3_2$	$0.5 * (\frac{1}{3})_1 + 0.5 * 0_2$	$0.5 * 1_1 + 0.5 * (\frac{1}{3})_2$	$0.5 * (\frac{5}{3})_1 + 0.5 * (\frac{10}{3})_2$
p_3	$2_1 + 1_2$	$0.5 * (\frac{1}{3})_1 + 0.5 * (\frac{1}{3})_2$	$0.5 * (\frac{1}{3})_1 + 0.5 * (\frac{1}{3})_2$	$0.5 * (2)_1 + 0.5 * (1)_2$
p_4	$3_1 + 1_2$	$0.5 * 0_1 + 0.5 * (\frac{1}{3})_2$	$0.5 * 0_1 + 0.5 * (\frac{1}{3})_2$	$0.5 * (4)_1 + 0.5 * (1)_2$
p_5	$1_1 + 2_2$	0	$0.5 * 0_1 + 0.5 * (\frac{1}{3})_2$	$0.5 * (3)_1 + 0.5 * (2.5)_2$
p_6	$2_1 + 2_2$	0	0	$0.5 * (4)_1 + 0.5 * (4)_2$

Supplementary Table 2: Ranking features based on predictions of sub_1 and sub_2. N_1 and N_2 denote the number of candidates with sub_1 and sub_2 extracted as substructures, respectively. The subscripts of the feature value $_1$ and $_2$ correspond to sub_1 and sub_2, respectively. The features are calculated among predictions of all substructures (Phase 1 and Phase 2), and unique substructures (Phase 2). We obtain Phase 1 predictions by removing incorrect SMILES, and Phase 2 predictions by further removing duplicated SMILES. Note that only the highest rankings are utilized to calculate the average ranking. r is the percentage for each substructure, i.e., $r_1 = \frac{N_1}{N_1+N_2}$, $r_2 = \frac{N_2}{N_1+N_2}$.

test data, with invalid reactions removed.

	All test set		Test set (invalid reactions removed)	
	Pair-wise ranker	Score function	Pair-wise ranker	Score function
Top-1	46.0	45.8	48.2	47.9
Top-2	56.2	56.0	58.8	58.6
Top-3	60.6	60.5	63.4	63.3
Top-4	63.1	63.1	66.0	66.0
Top-5	64.8	64.7	67.7	67.7
Top-6	65.9	65.9	69.0	69.0
Top-7	66.8	66.8	69.9	69.9
Top-8	67.5	67.5	70.6	70.6
Top-9	68.0	68.0	71.2	71.2
Top-10	68.5	68.5	71.6	71.6

Supplementary Table 3: The overall performance using the pair-wise ranking model and the score function.

The ranker slightly improves the accuracy of the top 1 to top 5. This is acceptable because, for most predictions, the percentages among top 1 and top 2 predictions will be 0. The results show that the improvements in our approach over the baselines are mainly attributed to the substructures and the inherent design of our approach. For the ranking model, we can achieve further improved performance by adding more ranking features, like top-3 percentage, top-4 percentage, etc.

Supplementary References

[1] Tetko, I. V., Karpov, P., Van Deursen, R. & Godin, G. State-of-the-art augmented nlp transformer models for direct and single-step retrosynthesis. *Nature communications* **11**, 1–11 (2020).
